# Ethnic and migration-related inequalities in health anxiety: A systematic review and meta-analysis

**DOI:** 10.3389/fpsyg.2022.960256

**Published:** 2022-08-26

**Authors:** Rieke Barbek, Sinje Henning, Julia Ludwig, Olaf von dem Knesebeck

**Affiliations:** Institute of Medical Sociology, University Medical Center Hamburg-Eppendorf, Hamburg, Germany

**Keywords:** migration, ethnicity, ethnic minority, health anxiety, hypochondriasis, health inequalities, systematic review, meta-analysis

## Abstract

**Background:**

Health anxiety exists on a continuum ranging from the absence of health awareness to the obsessive fear of having a serious illness despite reassurance. Its pathological manifestation can be diagnosed as hypochondriacal or illness anxiety or somatic symptom disorder. Health anxiety is associated with psychological distress and adverse life events, among others, and leads to considerable economic burden. Compared to the majority population, migrants, and ethnic minorities often face major health inequalities. Several mental illnesses and psychosomatic complaints are more common among these groups. To date, potential ethnic and migration-related inequalities in health anxiety have not been clearly described. However, they are of high relevance for the provision of adequate health care of this diverse and potentially vulnerable group. Thus, we conducted a systematic review and meta-analysis of health anxiety in migrants and ethnic minorities.

**Methods:**

A systematic literature search of PubMED, Web of Science, PsycINFO, and PSYNDEX was conducted, covering all studies published until 1^st^ of December 2021. Studies were selected if they employed validated measurement tools of health anxiety and examined migrants and/or ethnic minorities in comparison with the majority population. Meta-analytic methods were applied by using a random-effect model. The study quality was assessed with the Effective Public Health Practice Project Quality Assessment Tool (EPHPP).

**Results:**

We identified 18 studies from 445 studies initially screened. Of these, 14 studies conducted in North America with a total number of 5,082 study participants were included in the meta-analysis. The pooled effect size indicated a higher risk of health anxiety in migrants and ethnic minorities compared to the majority population (OR 1.39, 95%-CI 1.01–1.92). The results proved not to be robust according to publication bias (adjusted OR 1.18, 95%-CI 0.83–1.69) and fail-safe N (2/3 < benchmark *N* = 75) and are limited due to heterogeneity (*I*^2^ = 57%), small sample sizes and an overall low quality of included studies.

**Conclusion:**

To address the diversity of migrants and ethnic minorities, inter-sectional approaches across different countries are needed in research to shed further light on social inequalities in health anxiety linked to migration.

**Systematic review registration:**

PROSPERO, registration number CRD42022298458.

## Introduction

In 2020, 281 million people or 3.6% of the world population were classified as migrants (McAuliffe and Triandafyllidou, 2021). Compared to 2.3% in 1970, there is a clear upward trend which also contributes to the diversity within countries. Globally, the United States are the number one destination for migrants, whereas India and Mexico are the number one countries to be left (McAuliffe and Triandafyllidou, 2021). Migration is understood as the temporary movement from the country of birth to the country of residence and immigration refers to a permanent movement. Reasons for migration are mostly due to work, family, or study. Refugees, however, are forced to leave their country due to e.g., political conflicts or disasters and are highly vulnerable. The term ethnicity (former race) ascribes people to a certain group by characteristics like origin, religion, or language (World Health Organization, 2010b; Bhopal, 2014). While the concepts of ethnicity and race are widely used in North America, migration background is frequently used in Europe. Since migration and ethnicity are related social constructs and often overlap, the World Health Organization calls for a joint consideration of both in research (World Health Organization, 2010b). Nevertheless, people with a migration background are a heterogeneous group regarding e.g., socio-demographic aspects, the immigrant generation or the length of stay (Rommel et al., 2015).

According to the concept of social determinants of health (Marmot and Wilkinson, 2011; World Health Organization, 2022b), experiences during migration process as well as discrimination are important for health and wellbeing. Hence, a major topic in public health research is the question to what extent health inequalities exist between migrants or ethnic minorities and the majority population (Davies et al., 2006; Chung and Griffiths, 2018). Since migrants are often not adequately represented in data on population health, and some groups are still not represented at all (World Health Organization, 2010b), research is limited. Until now, the findings tend to be quite heterogeneous. For example, migration is associated with higher rates of diabetes, specific infectious diseases, and post-traumatic stress disorder, the latter two especially occurring in refugees (Hunter and Schmidt, 2010; World Health Organization, 2010b). Whereas, e.g., allergies or alcohol misuse are less often detected in migrants (Rommel et al., 2015; World Health Organization, 2021). Migrants even tend to be among the healthiest in their home country, which is described as the healthy migrant effect (World Health Organization, 2010a; Bhopal, 2014). However, in the country of residence, refugees, migrants and several ethnic minority groups often face psychosocial, behavioral and material deprivation, like discrimination, poor living and working conditions, and inadequate access to health care services (World Health Organization, 2022a). Further, there is evidence that health services are used less in these groups (World Health Organization, 2010b; Klein and von dem Knesebeck, 2018). This might explain a frequently higher vulnerability of these groups regarding certain somatic or mental illnesses, e.g., stress-related and somatoform disorders which also includes (pathological) health anxiety.

Health anxiety is defined as the “preoccupation with the fear of having a serious disease, which persists in spite of appropriate medical reassurance” (Muse et al., 2010). Health anxiety exists on a continuum ranging from the absence of health awareness to excessive concerns about one's own health status (Ferguson, 2009; Bailer et al., 2016). Severe health anxiety in a pathological manner can be diagnosed as hypochondriacal disorder or hypochondriasis, illness anxiety disorder (IAD) or somatic symptom disorder (SSD) (Muse et al., 2010; American Psychiatric Association, 2013; World Health Organization, 2019). It is assessed either with diagnostic instruments or diagnostic interviews (Tyrer, 2018). However, appropriate cut-off scores to identify individuals with severe health anxiety are still under discussion (Creed and Barsky, 2004; Weck et al., 2014; Hedman et al., 2015). Health anxiety as a broader concept is present in about 10% of the general population and over 30% in mental health care settings (Weck et al., 2014). The prevalence for pathological health anxiety, primarily hypochondriasis, is <1% (Weck et al., 2014). To date, only few studies examine the newly defined diagnoses of IAD and SSD with prevalence rates of 7% for IAD in an Indian medical sample (Pandey et al., 2017) and almost 34% in case of SSD in Chinese patients (Cao et al., 2020). Health anxiety is associated with increased psychosomatic complaints and mental comorbidities, such as depression or anxiety disorders, (Newby et al., 2017) and leads to a reduction in health-related quality of life (Bleichhardt and Hiller, 2007). The economic burden of health anxiety is considerable, as it is associated with an increase in health care use (Barsky et al., 2001; Newby et al., 2017; Norbye et al., 2022), extended sick-leave (Eilenberg et al., 2015), and disability (Mykletun et al., 2009).

The etiology of health anxiety is explained in the cognitive-behavioral model of Barsky and Wyshak (1990) and the model of somatosensory amplification of Warwick and Salkovskis (1990). According to these models, negative and traumatic life events during childhood (e.g., a serious illness or abuse) can lead to dysfunctional illness responses and catastrophizing. Acute stressors which are often faced by refugees and certain migrant groups pre, during, and post migration process (e.g., the death of a relative, discrimination, or material deprivation) can activate these catastrophizing tendencies and lead to negative assumptions of the body (Hunter and Schmidt, 2010; World Health Organization, 2010a; Mölsä et al., 2014). Even slight symptoms or bodily sensations are then misinterpreted as a threatening illness (Sauer and Witthöft, 2020). Furthermore, negative treatment experiences like language barriers or cultural differences in illness perceptions can maintain or worsen the misinterpretation. As a result, (pathological) health anxiety can occur (Haasen, 2000; Arbisi et al., 2002; Ekblad, 2020), increasing inequalities in the particular vulnerable groups of migrants and ethnic minorities (World Health Organization, 2010b).

A meta-analysis by Barbek et al. (2021) found a higher risk of health anxiety in low socio-economic individuals, indicating social inequalities in health anxiety on a vertical level. Still, social inequalities in health anxiety according to migration background are not well studied and remain inconclusive (Mölsä et al., 2014; Weck et al., 2014; Akariya et al., 2021). Therefore, we examine differences in health anxiety between migrants and ethnic minorities compared to the majority population in the respective countries in a systematic review and meta-analysis. These findings may help to understand the specific needs and improve the health care of particular vulnerable groups of migrants and ethnic minorities.

## Methods

### Search strategy

According to the population, exposure and outcome criteria (PEO) (Pollock and Berge, 2018), the search strategy consisted of the following terms with closely related words: health anxiety, health worries, hypochondriasis, illness anxiety (disorder), somatic symptom disorder (as outcome) in combination with migration, immigrant, ethnicity, race, refugee, minority (as exposure in any adult population; for the full search strategy see Supplementary data 1). The following four databases were searched for relevant studies: PubMED, Web of Science, PsycINFO, and PSYNDEX. Additionally, the reference lists of included studies were screened.

### Study selection

Review team consisted of three reviewers (RB; SH; JL). The results were processed in the Citavi reference management system. After removing duplicates, RB and JL independently screened all titles and abstracts and RB and SH independently reviewed all potential full-texts in concordance with pre-defined inclusion and exclusion criteria. Disagreement was solved *via* discussion in the review team. Inclusion criteria consisted of the following: (a) empirical studies published until 1^st^ of December 2021; (b) full text in English or German; (c) adult population; (d) health anxiety assessed with a validated self-rating scale or diagnostic interview (criteria for validation were either the psychometric analysis of the assessment tool or a cross-reference to a validation study); (e) comparing a population of migrants, immigrants, refugees, or ethnic minority with the majority population in one country. The following exclusion criteria were defined: (a) qualitative studies, reviews or meta-analyses, and methodical studies on measurement invariance; (b) specific forms of health anxiety (e.g., fear of progression, modern health worries, COVID-related anxiety).

### Extraction of study data and meta-analytical procedure

Two reviewers (RB; SH) independently extracted the following study characteristics: study design, country, setting/population, sample size, assessment of health anxiety, comparators, quality assessment, and summarized results. For the latter, univariate or adjusted results, if reported, were categorized as follows: significant or non-significant differences in health anxiety between migration or ethnic minority population and majority population. Since the majority of included studies were conducted in North America (USA and Canada), only these studies were pooled for the meta-analysis to reduce heterogeneity. To calculate the pooled effect size, prevalence rates were converted into log odds ratios (OR) with standard error (SE). In case of mean values, these were first transformed into standardized mean differences and second into log OR with SE (Lenhard and Lenhard, 2016; Borenstein et al., 2021). Since none of the studies reported adjusted models suitable for meta-analytical calculation, the meta-analysis includes only univariate results. Although study authors were contacted in case of missing data, none was able to provide the requested information. Hence, missing data was imputed by calculating the mean of the effect estimate and/or SE of the remaining studies. Due to great variety in observational study designs, the random effect model was chosen for meta-analysis (Hedges and Vevea, 1998). The standardized effect measures were weighted according to their SE. To assess the extent of heterogeneity, Cochran's Q and Higgins *I*^2^ were calculated (Higgins and Thompson, 2002; Harrer et al., 2021). Furthermore, a sensitivity analysis was conducted to examine pre-defined moderators of the pooled effect size (Aromataris and Munn, 2020). Stratified meta-analyses were conducted for each of the following moderators: assessment of health anxiety, comparators, study population, and study quality. To determine the risk of publication bias, Egger's regression test (no risk if *p* > 0.05) and the trim-and-fill method were applied (Egger et al., 1997; Duval and Tweedie, 2000). Further methods included the fail-safe N to test the stability of our meta-analysis. Therefore, fail-safe Ns with different preconditions were assumed (insignificant α 0.05 and 0.1; irrelevant OR 0.95; stable results if fail-safe N>5n+10) (Rosenthal, 1979; Orwin, 1983; Viechtbauer, 2021). The meta-analysis was calculated and visualized with the *R* packages “esc”, “meta”, “metafor", and “tidyverse”. Effect sizes were pre-calculated with the internet-based calculation tool Psychometrica (Lenhard and Lenhard, 2016; Harrer et al., 2021). For the *R* syntax see Supplementary data 2.

### Quality assessment with the EPHPP

To determine the study quality, two reviewers (RB; SH) independently applied the Effective Public Health Practice Project Quality Assessment Tool (EPHPP) (Thomas et al., 2004). The EPHPP is a compact and validated tool suitable for interventional and observational studies. It consists of six categories: selection bias, study design, confounders, blinding, data collection method, and withdrawals/dropouts. Each category is rated as strong, moderate, or weak. The total score is calculated based on the number of weak categories (strong = 0 weak categories; moderate = 1 weak category; weak = more than 1 weak category) (Thomas et al., 2004). Since our systematic review consisted of observational studies only, the EPHPP was slightly adapted to the quality rating standards for observational studies as stated by the Cochrane Collaboration (Armijo-Olivo et al., 2012; Higgins et al., 2022). First, the category blinding was dropped because it refers to interventional studies only. Instead, the category statistical analysis was included. Second, cross-sectional studies were rated as moderate, as they are suitable for answering associative questions; cohort and case-control studies were rated as strong. Third, the focus of confounders was widened instead of rating pre-interventional group differences only. Fourth, withdrawals and dropouts were completed by loss to follow-up regarding cohort studies. The adapted tool has already proven its usefulness in a previous review of the research group (Barbek et al., 2021).

We conducted our systematic review and meta-analysis in concordance with the PRISMA standards (Moher et al., 2009) and MOOSE statement (Stroup et al., 2000) (see Supplementary Table 1). The review protocol was registered at PROSPERO under the number CRD42022298458.

## Results

### Screening and inclusion of studies

**Figure 1:**
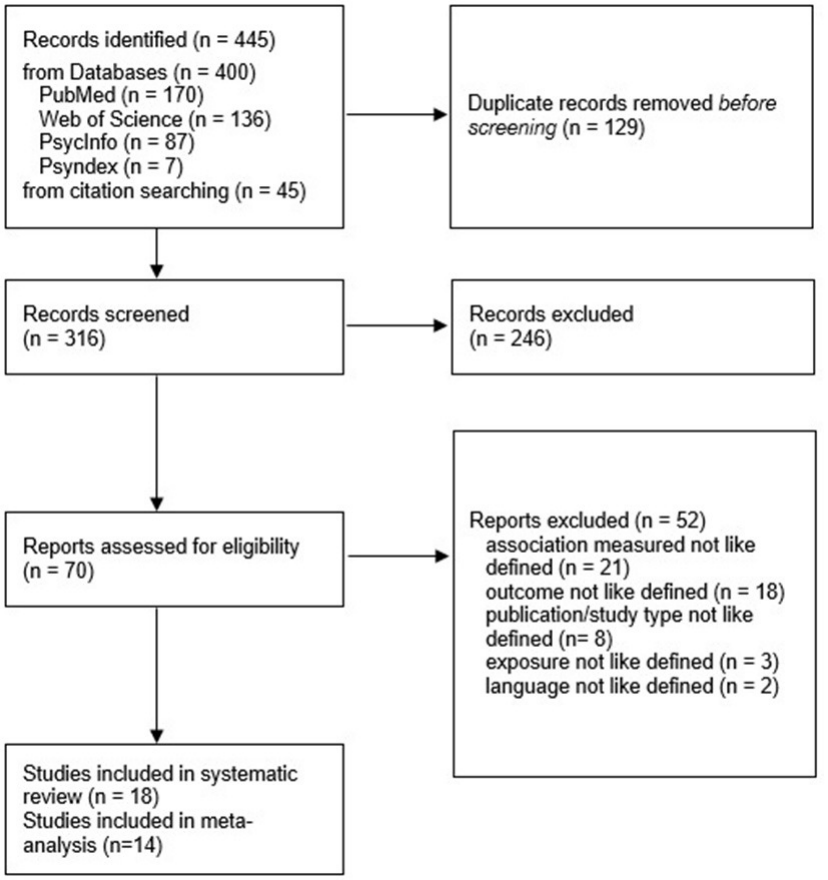
PRISMA 2020 flow diagram. From: Page et al. (2021).

We identified 400 records through database screening and additional 45 records from citation searching, resulting in a total number of 445 records. After removing duplicates, we screened 316 titles and abstracts. From the remaining 70 full texts, we excluded 52 records due to our pre-defined inclusion and exclusion criteria. Main reasons for exclusion were: lack of the association of interest, outcome or exposure not like defined, wrong publication or study type, not written in English or German. Finally, we included 18 studies in our systematic review. Of these, we compiled a subsample of 14 studies from North America for our meta-analysis (see Figure 1). During the screening process, our inter-rater reliability reached 90%. For the remaining 10% of studies, we discussed their suitability until agreement. The details of excluded studies and the reason for exclusion can be found in the Supplementary Table 2.

Table 1 presents the main study characteristics and summarized results of the 18 included studies with a total number of 5,914 study participants in our systematic review. The majority was of cross sectional design (*n* = 13), whereas case-control studies (*n* = 3) and cohort studies (*n* = 2) were less represented. More than three-quarters of studies were conducted in North America (*n* = 14), three studies in Europe and one study in Asia. In terms of the study population/setting, half of the studies investigated the general (or a specific) population (*n* = 9) and patients (either general or mental health care setting) (*n* = 9), respectively. Regarding the assessment of health anxiety, two-thirds of studies employed validated self-rating scales (*n* = 12), the remaining one-third used standardized diagnostic interviews (*n* = 6). Furthermore, the majority of included studies compared ethnic minority groups with the majority population (*n* = 14). Ethnic minorities were mainly of Black, Hispanic, or Asian background (according to race, ethnicity, or nationality as stated in the included studies). Only four studies explicitly investigated a population of immigrants (all of Hispanic or Asian background) or refugees (Somalis), who all had own migration experiences. Notably, at least one-quarter was of moderate study quality (*n* = 5) but three-quarters of weak quality (*n* = 13). Detailed results of the quality assessment can be found in Supplementary Table 3. The inter-rater reliability of the quality assessment reached 85%. The remaining 15% of disagreement were solved *via* discussion.

**Table 1:** Study characteristics and summarized results of included studies (*n* = 18 studies, with *n* = 5,914 study participants).

**#**	**Author (year)**	**Study design**	**Country**	**Population/setting**	**Sample size**	**Assessment of health anxiety**	**Comparators**	**Quality Assessment**	**Summarized results^d, e^**
1	Akariya et al. (2021)	Cross-sectional	Israel	Jewish and Arab Israeli young adults	305	Short Health Anxiety Inventory (SHAI)	Arabs^a^/Jews^c^	Moderate	n.s.^f^
2	Barsky et al. (1998)*	Cohort	United States	Patients of a primary care clinic	186	Structured diagnostic interview for hypochondriasis based on DSM-III-R criteria	Black^a^/Asian^a^/White^c^	Weak	Cohort 1: s. (Black>White) Cohort 2: n.s.
3	Barsky et al. (1990)*	Cross-sectional	United States	Outpatients of a general medical clinic	116	Structured diagnostic interview for hypochondriasis based on DSM-III-R criteria	Black^a^/Asian^a^/White^c^	Weak	s. (Black>White)
4	Bhatt et al. (1989)	Cross-sectional	United Kingdom	Patients of general practitioners	150	Illness Behavior Questionnaire (IBQ)	Gujarati^a^/Urdu^a^/English^c^ (Language)	Weak	s. (Gujarati> Urdu>English)
5	Bravo and Arrufat (2005)*	Case-control	United States	Older adults with and without depression	106	Illness Attitude Scale (IAS), Yes-No format	Hispanic immigrants^b^/non-Hispanic^c^	Weak	s. (Hispanic> non-Hispanic)
6	Escobar (1998)*	Cross-sectional	United States	Outpatients of primary care services	1,456	Composite International Diagnostic Interview (CIDI)	Hispanic immigrants^b^/US-born^c^	Weak	n.s.
7	Fergus et al. (2017)*	Cross-sectional	United States	Patients of a community health center	538	Whiteley Index (WI-6)	Black^a^/Hispanic^a^/Asian^a^/ non-Hispanic White^c^	Moderate	n.s.
8	Gerdes et al. (1996)*	Cross-sectional	United States	Outpatients of an outpatient medicine clinic	210	Whiteley Index (WI-14), modified version	non-White^a^/ White^c^	Weak	n.s.
9	Goel et al. (2002)*	Cross-sectional	United States	Research volunteers of light or negative air ion treatment	165	Structured Interview Guide for the Hamilton Depression Rating Scale – Seasonal Affective Disorder (SIGH-SAD)	Black^a^/White^c^	Moderate	not reported (Black>White)
10	Hollifield et al. (1999)*	Cross-sectional	United States	Patients from a family practice center	185	Illness Attitudes Scale (IAS)	Hispanic^a^/White^c^	Weak	n.s.
11	Kibbey et al. (2021)*	Cross-sectional	United States	Undergraduate students	641	Short Health Anxiety Inventory (SHAI)	Black^a^/Hispanic^a^/Asian^a^/ White^c^	Moderate	n.s.
12	Looper and Kirmayer (2001)*	Cross-sectional	Canada	Community sample (telephone survey)	576	Composite International Diagnostic Interview (CIDI)	Asian and Hispanic immigrants^b^/Canadian-born^c^	Weak	n.s.
13	Mölsä et al. (2014)	Case-control	Finland	Older Somali refugees and Finns from Helsinki	256	Whiteley Index (WI-7)	Somali refugees^b^/Finns^c^	Weak	s. (Finns > Somalis)
14	Noyes et al. (2005)*	Cross-sectional	United States	Nationwide telephone survey	937	Composite International Diagnostic Interview (CIDI); Illness Worry Scale (IWS)	non-White^a^/ White^c^	Weak	n.s.
15	Noyes et al. (2004)*	Cohort	United States	Gulf War veterans	602	Whiteley Index (WI-14)	Black^a^/White^c^	Weak	n.s.
16	Noyes et al. (1999)*	Cross-sectional	United States	Relatives of (non-) hypochondriacal probands	169	Whiteley Index (WI-14)	non-White^a^/White^c^	Weak	n.s.
17	Pilowsky and Spence (1977)	Case-control	Australia	Patients of general practitioners	134	Illness Behavior Questionnaire (IBQ)	Greek^a^/Anglo-Greek^a^/Anglo-Saxon^c^ (language)	Moderate	n.s.^g^
18	Pine (1983)*	Case-control	United States	Urbanized obese and non-obese volunteers	160	Mini-Mult Minnesota Multiphasic Personality Inventory (MMPI)	American Indian^a^/ Caucasian^c^	Weak	n.s.

n.s., not significant on α < 0.05 level; s., significant on α < 0.05 level.

*study included in meta-analysis.

^a^ethnic minority; ^b^immigrants; ^c^majority population; ^d^risk for/rate of health anxiety between comparators. When both, univariate and multivariate analysis have been performed, the results of the multivariate model are reported; ^e^adjusted for sex, age, self-criticism, depression, anxiety sensitivity, alexithymia and stressful life events; ^f^adjusted for age and sex.

For a first overview, we summarized the data as reported in the included studies (Table 1). Four of the included studies reported a significantly higher risk or rate of health anxiety in migrants or ethnic minorities compared to the majority population. Another study also reported this difference, but the information on the level of significance was missing. Only one study stated a significant inverse association, whereas two-thirds of studies found no significant association (*n* = 12). Two studies presented results of multivariate models adjusted for age and gender, in one case, analyses additionally controlled for several psycho-social variables. The remaining 16 studies published only non-adjusted results.

### Meta-analytical results

**Figure 2:**
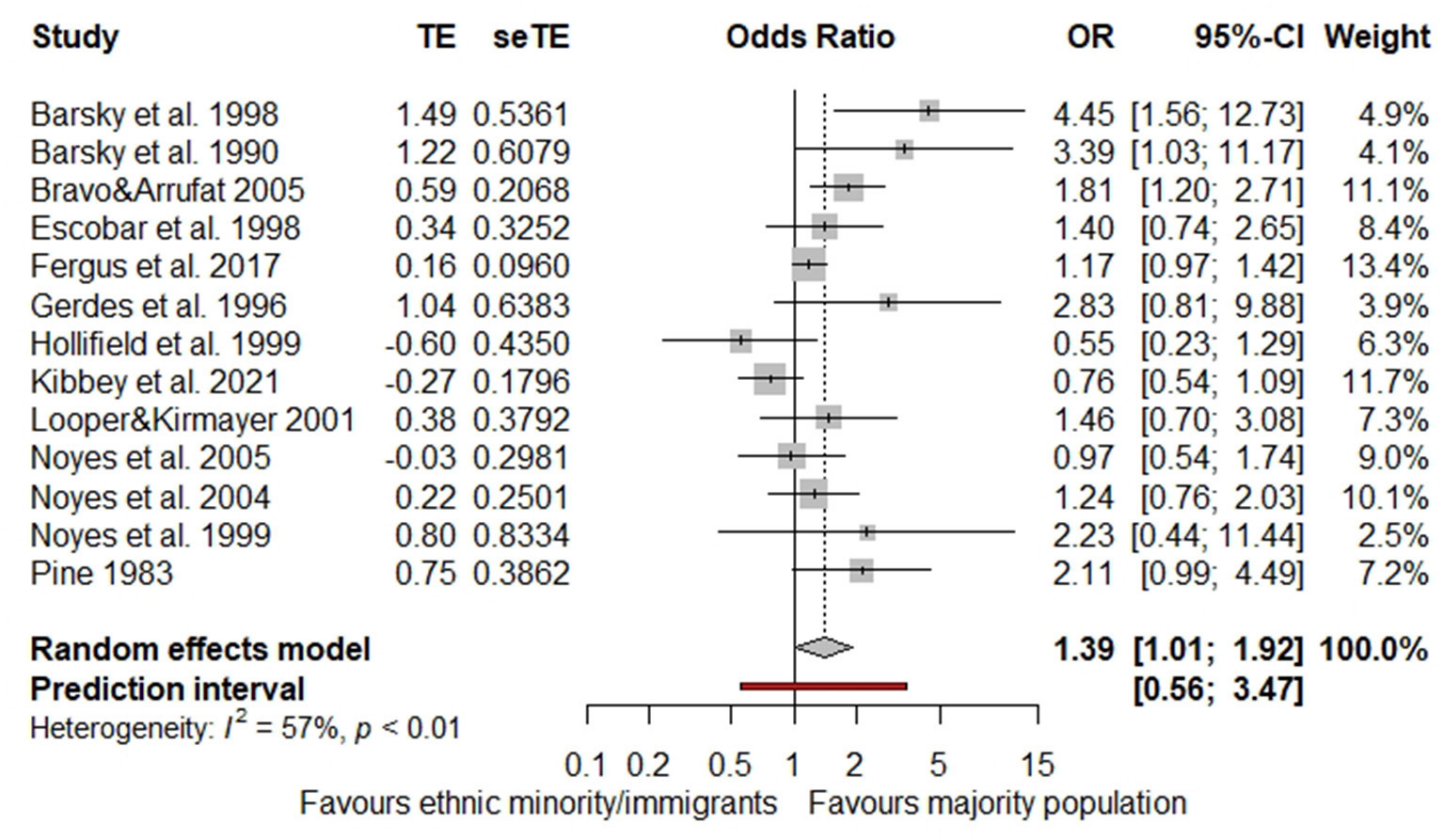
Pooled effect size of studies from North America (*n* = 13 studies, with *n* = 4,926 study participants, outlier-adjusted).

In our meta-analysis, we analyzed all 14 studies conducted in North America (USA *n* = 13; Canada *n* = 1) with a total number of 5,082 study participants. We calculated log ORs based on prevalence rates for nine studies and for four studies based on standardized mean differences. For the remaining study, we imputed values due to missing data. Since the standardized effect measure of one study proved oneself as an outlier, we only report the results of the remaining 13 studies. According to our model, migrants and ethnic minorities had a significant, almost 40% higher risk of health anxiety compared to the majority population (OR 1.39, 95%-CI 1.01–1.92; see Figure 2). However, results were significant in only three of the 13 studies. In seven studies, the risk was higher but insignificant and the remaining three studies found an insignificant inverse risk.

### Sensitivity analysis

In the stratified analyses, we found no significant differences between the subgroups (Table 2; *p* of all subgroups > 0.05). This indicates that none of the pre-defined criteria are relevant moderators. Nonetheless, some indications can be drawn from our results. The risk of health anxiety in migrants and ethnic minorities was higher when assessed in patients (either general or mental health care setting) compared to population-based studies (OR 1.59, 95%-CI 0.89–2.84). Also, the risk of health anxiety in migrants and ethnic minorities was higher when assessed with a validated diagnostic interview compared to self-rating scales (OR 1.69, 95%-CI 0.80–3.59). Furthermore, immigrants formed a homogenous subsample with a slightly higher risk of health anxiety compared to the overall effect size (OR 1.57, 95%-CI 1.10–2.24).

**Table 2:** Sensitivity analysis of studies from North America (*n* = 13 studies, with *n* = 4,926 study participants, outlier-adjusted).

	***N***	**OR (95%-CI), I^2^**	***p*_subgroup_**
**Overall**	13	1.39 (1.01–1.92), 57%	
**Assessment of health anxiety**			0.361
Diagnostic interview	5	1.69 (0.80–3.59), 51%	
Self-rating scale	8	1.26 (0.83–1.90), 61%	
**Comparators**			0.465
Immigrants	3	1.57 (1.10–2.24), 0%	
Ethnic minorities	10	1.35 (0.86–2.10), 61%	
**Study population**			0.335
Patients (general or mental health care setting)	7	1.59 (0.89–2.84), 64%	
Population (general or specific)	6	1.21 (0.79–1.83), 41%	
**Study quality**			0.071
Strong	0	/	
Moderate	2	0.96 (0.06–14.65), 78%	
Weak	11	1.56 (1.09–2.24), 41%	

CI, confidence interval; OR, odds ratio.

### Publication bias and stability of meta-analysis

The heterogeneity between studies was moderate, with Higgins *I*^2^ = 57%. Although stratification reduced heterogeneity in some cases, no clear trend emerged (Table 2). Only the subsample of immigrants was homogenous (*I*^2^ = 0%). Second, we evaluated potential publication bias. Egger's regression test gave no indication for biased results (intercept −0.03, *t* = 1.64, *p* = 0.130). Otherwise, after imputing studies to adjust for potential publication bias, the pooled OR decreased and became insignificant (studies trimmed *n* = 4, adjusted OR 1.18, 95%-CI 0.83–1.69). For visualization, the adjusted funnel plot is presented in the Supplementary Figure 1. Third, we calculated different fail-safe Ns to examine the effect of insignificant unpublished data on our meta-analysis. Two out of three fail-safe Ns did not reach the benchmark (α 0.05 fail-safe *N* = 57; α 0.1 fail-safe *N* = 103; OR 0.95 fail-safe *N* = 38; with benchmark 5x13+10 = 75). Taken together, results for publication bias and stability of our meta-analysis are inconclusive, questioning the robustness of the meta-analytical results.

## Discussion

### Summary and interpretation in context of literature

To the best of our knowledge, we conducted the first systematic review investigating ethnic and migration-related differences in health anxiety. Since migration and ethnic minority groups overlap, especially in the USA, we studied these groups together, as suggested (Haasen, 2000; World Health Organization, 2010b). To reduce heterogeneity, we included only the 14 studies conducted in North America with more than 5,000 individuals in our meta-analysis. The overall pooled effect size indicated a higher risk of health anxiety in migrants and ethnic minorities. However, summarizing Egger's regression test, the trim-and-fill method, and the fail-safe N, results on the robustness of our meta-analysis were inconclusive. Reason might be the small number and sample sizes of included studies. In our sensitivity analysis, none of the pre-defined moderators, namely assessment of health anxiety, comparators, study population, and study quality, revealed a significant influence on the association under study. Regarding the heterogeneity, only the three studies analyzing immigrants (all of Hispanic or Asian background) formed a homogeneous subgroup with a slightly increased risk of health anxiety. Overall, test for publication bias and sensitivity analysis revealed inconsistent results. Accordingly, further research is needed to shed light on migration-related differences in health anxiety with a focus on the diversity of the groups of migrants, refugees, and ethnic minorities.

Ethnic and migration-related inequalities in health anxiety can be explained following the concept of social determinants of health (Marmot and Wilkinson, 2011; World Health Organization, 2022b). According to this concept, migrants often face negative and traumatic life events pre, during and post-migration, acute psychosocial stressors, behavioral and material deprivation as well as negative treatment experience in the health care setting. Since the etiology of health anxiety, especially in its pathological extent, is strongly related to negative life events and acute stressors (Barsky and Wyshak, 1990), the possible onset or maintenance of health anxiety in some minority populations can be assumed. However, since our meta-analytical results were inconclusive and several included studies found no ethnic or migration-related disparities in health anxiety, further investigation is needed. Like stated e.g., by Kibbey et al. (2021), especially the high heterogeneity of people with migration background or of different ethnicities might not have been adequately represented in the included studies. This is in line with our sensitivity analysis, indicating a higher risk of health anxiety in the homogeneous group of Hispanic or Asian immigrants residing in the United States but not for the heterogeneous group of ethnic minorities including e.g., Black, Asian, Hispanic, Indian Americans, and other unspecified non-White residents in the United States and Canada. Accordingly, a generalization for all groups of migrants or ethnic minorities is not possible. To what extent health anxiety is present e.g., in Turkish migrants living in Germany, is still unknown. Hence, further research is needed which explicitly examines health anxiety in different migration populations and settings accounting for the heterogeneity of migration and ethnic minority populations as well as the contextual factors in the different countries of residence. Thereby, inter-sectional approaches and multivariate models can shed light on the potential interaction of different socio-demographic variables in the context of health anxiety (Crenshaw, 1989; Kibbey et al., 2021). Additionally, research is needed to shed light on the discrepancy of the generally lower health care utilization among migrants and the higher health care utilization in people with health anxiety. Overall, it remains an open question whether ethnic and migration-related inequalities in health anxiety exist in a systematic and consistent manner.

### Limitations

The following methodological aspects should be considered when interpreting our results. First, our analyses are limited due to the small number of studies identified through the systematic literature search. This reflects the need for further research in the under-represented area of inequalities in mental health among migrants and minority groups. The small sample size also affects the sensitivity analysis, since subgroups should include *n* > 5 studies for reliable results (Borenstein et al., 2021). Accordingly, the results can only give indications which need further confirmation. Additionally, the small sample sizes of the respective study populations can be seen as one reason why the majority of included studies did not find significant ethnic or migration-related differences in health anxiety contrary to the overall effect size of our meta-analysis. Second, the overall low quality of the included studies limits our results. The studies were limited to answer our research question mainly due to the cross-sectional design and a lack of multivariate analysis. Future research in this field should provide adjusted models to increase the quality and reliability of results. Third, heterogeneity of included studies limits our findings which has several reasons: The included studies assessed migration and ethnic minority status with a variety of variables like migration/immigration, refugee status, ethnicity/race, nationality, or language. Based on this data, we were not able to satisfactorily differentiate between people with and without own migration experiences. Future research should collect both, migration background and ethnicity to reflect the high diversity of these groups. Together with a great variety of assessment tools for health anxiety, the included studies were even more heterogeneous. The heterogeneity also remained in the sensitivity analysis. Accordingly, other moderators besides the ones we assumed based on theoretical considerations could be of relevance to further modify the potential association between migration and health anxiety. Fourth, the inclusion of studies only written in English or German might have led to selection bias. However, we rated this risk as low, since most of the studies identified were conducted in English-speaking countries, mostly North America. The large number of studies conducted in North America might be explained by the fact that Mexico–United States is the number one country-to-country corridor worldwide (McAuliffe and Triandafyllidou, 2021). However, our results are not generalizable to other countries or cultures, since the situation of migrants, refugees and ethnic minorities is highly contextual (e.g., depending on the health care system, legal rights, or the integrational approaches made in the country of residence). Accordingly, further research in different countries with diverse cultural approaches is needed.

## Conclusion

Research on mental health in migrant and ethnic minority populations is lacking, limited to certain countries and hence not generalizable. This is also the case for health anxiety, since our systematic review and meta-analysis found few suitable studies with high heterogeneity revealing no consistent ethnic and migration-related inequalities in health anxiety so far. However, clear insights are essential to adequately respond to the needs of these diverse groups. Since migration experiences are often related to high psychological stressors and behavioral as well as material deprivation, migrants and other minority groups might be particularly vulnerable to several mental illnesses, including (pathological) health anxiety. Health anxiety needs to be recognized early and appropriately treated in health care to reduce individual and economic burdens. Intersectional approaches and multivariate analysis can help to gain a deeper understanding of the associations between health anxiety, (psychological) distress, and all kinds of discrimination or deprivation due to migration, ethnicity and other social determinants like gender, age and socio-economic status. Additionally, since our sensitivity analysis indicated only specific immigrants as a homogeneous subgroup, future attempts should consider the heterogeneousness of migration and ethnic minority populations as well as the contextual factors in the country of residence (e.g., the health care system or legal aspects) to obtain reliable results.

## Data availability statement

The datasets/overview of studies used and analyzed during the current study are available from the corresponding author on reasonable request.

## Author contributions

RB developed the research question, performed the screening, quality assessment, analysis and interpretation of data, and was a major contributor in writing the manuscript. SH performed the screening, quality assessment, and analysis of data and was a contributor in writing the manuscript. JL performed the screening and was a contributor in writing the manuscript. OK developed the research question and was a contributor in writing the manuscript. All authors read and approved the final manuscript.

## Funding

The analyses are part of the project Social Inequalities in Aggravating Factors of Somatic Symptom Persistence which is part of the Research Unit 5211 (RU 5211) Persistent SOMAtic Symptoms ACROSS Diseases: From Risk Factors to Modification (SOMACROSS), funded by the German Research Foundation (Deutsche Forschungsgemeinschaft, DFG, project number 445297796, speaker: Professor Bernd Löwe, MD); see also https://gepris.dfg.de/gepris/projekt/445297796.

## Conflict of interest

The authors declare that the research was conducted in the absence of any commercial or financial relationships that could be construed as a potential conflict of interest.

## Publisher's note

All claims expressed in this article are solely those of the authors and do not necessarily represent those of their affiliated organizations, or those of the publisher, the editors and the reviewers. Any product that may be evaluated in this article, or claim that may be made by its manufacturer, is not guaranteed or endorsed by the publisher.
