# Supplementary material for: Ethnic and migration-related inequalities in health anxiety: A systematic review and meta-analysis
Source: Front Psychol. 2022 Aug 26;13:960256. doi: 10.3389/fpsyg.2022.960256 (PMC9462455; doi:10.3389/fpsyg.2022.960256)

Supplementary Material

1 Supplementary Data

1.1 Supplementary Data 1: Search strategy

("health anxi*"[tw] OR "health worr*"[tw] OR "health fear*"[tw] OR "health-related fear*"[tw] OR "illness worr*"[tw] OR "illness fear*"[tw] OR "illness concern*"[tw] OR hypochondri*[tw] OR "illness anxiet*"[tw] OR "somatic symptom disorder*"[tw])

AND

(migrat*[tw] OR migrant*[tw] OR immigrant*[tw] OR ethnic*[tw] OR race[tw] OR citizenship*[tw] OR foreigner*[tw] OR "foreign born"[tw] OR "foreign population"[tw] OR nationality[tw] OR "asylum seeker*"[tw] OR refugee*[tw] OR "country of origin"[tw] OR "country of birth"[tw] OR "cultural group*"[tw] OR "ethnocultural group*"[tw] OR "marginal group*"[tw] OR "marginalized group*"[tw] OR "disadvantaged group*"[tw] OR minorit*[tw])

1.2 Supplementary Data 2: R syntax

```
######meta-analysis to measure social inequalities in health anxiety######

#####syntax#####

####initial preparation####

#istall packages (once)#

install.packages("tidyverse")

install.packages("meta")

install.packages("metafor")

#load packages#

library(tidyverse)

library(meta)

library(metacor)

library(metafor)

###convert effect sizes###

install.packages("esc")
```

```
library(esc)

library(easystats)

#convert d to OR - with esc#

convert_d2or(0.119, 0.2388, 602, es.type = "logit")  #Noyes'04#

convert_d2or(0.088, 0.0916, 533, es.type = "logit")  #Fergus'17#

convert_d2or(-0.342, 0.1254, 256, es.type = "logit")  #Mölsä'14#

convert_d2or(1.414, 0.2083, 134, es.type = "logit")  #Pilowsky'77#

convert_d2or(0.974, 0.1792, 137, es.type = "logit")  #Bhatt'89#

convert_d2or(0.547, 0.1999, 106, es.type = "logit")  #Bravo'05#

convert_d2or(2.18, 0.1739, 156, es.type = "logit")  #Goel'02#

###meta-analysis - subsample consisting only of studies from north america (USA&Canada) without Outlier Goel'2002###

#import dataset from excel#

library(readxl)

data_OR_USA_outl <- read_excel("data_OR_USA_outl.xlsx")

View(data_OR_USA_outl)

#assign correct format to variables#

data_OR_USA_outl$logOR<-as.numeric(data_OR_USA_outl$logOR)

data_OR_USA_outl$SElogOR<-as.numeric(data_OR_USA_outl$SElogOR)

#metagen#

m.gen <- metagen(TE = logOR,

        seTE = SElogOR,

        studlab = author,

        data = data_OR_USA_outl,

        sm = "OR",

        fixed = FALSE,
```

```
random = TRUE,
method.tau = "PM",
hakn = TRUE,
title = "meta-analysis subsample north america - outlier-adjusted")
m.gen
#forest plot#
forest.meta(m.gen,
prediction=TRUE,
label.left="Favours ethnic minority/immigrants",
label.right="Favours majority population",
xlim = c(0.1,15),
print.tau2=FALSE,
leftlabs="Study")
##sensitivity analysis##
#Egger's test for publication bias; p>0.05 no indication for publication bias#
metabias(m.gen, method.bias="linreg")
#funnel plot#
funnel.meta(m.gen)
#funnel plot with trim-and-fill-method#
taf_r <- trimfill(m.gen)
summary(taf_r)
funnel(taf_r, legend=TRUE)
#subgroup analysis#
update.meta(m.gen, subgroup=HA_measure, tau.common=TRUE)
update.meta(m.gen, subgroup=EM_measure, tau.common=TRUE)
update.meta(m.gen, subgroup=study_population, tau.common=TRUE)
```

```
update.meta(m.gen, subgroup=study_type, tau.common=TRUE)

update.meta(m.gen, subgroup=study_quality, tau.common=TRUE, control=list(stepadj=0.5,
maxiter=1000))

###further sensitivity analysis###

##influence analysis##

#meta-analysis with the package metafor#

logOR <- rma.uni(yi=logOR,

        sei=SElogOR,

        data=data_OR_USA_outl,

        slab=author,

        method="PM",

        test="knha",

        measure="OR",

        digits=2)

logOR

#exponentiation of logarithmic estimate and CI#

exp(0.33)

exp(0.01)

exp(0.65)

#CI of heterogeneity measures#

confint(logOR)

#standardized residuals#

rstandard(logOR)

res <- rstudent(logOR)

res

#influencing cases and leave-one-out#
```

```
inf <- influence(logOR)

inf

plot(inf, plotdfb = TRUE)

leav <- leave1out(logOR, transf = exp, digits = 2)

leav

#fail-safe N - Rosenthal alpha=0.05, 0.1; Orwin target~OR=0.95->logOR=-0.051; stable meta-analytical results if calculated Nfs > benchmark 5n+10 ~ 75#

fsn(yi=logOR,

   sei=SElogOR,

   data=data_OR_USA_outl,

   type="Rosenthal",

   alpha=0.1,

   digits=2)

fsn(yi=logOR,

   sei=SElogOR,

   data=data_OR_USA_outl,

   type="Orwin",

   target=-0.051,

   weighted=TRUE,

   digits=2)

####end####
```

2 Supplementary Tables and Figures

2.1 Supplementary Table 1: MOOSE Checklist for Meta-analyses of Observational Studies

Item No	Recommendation	Reported on Manuscript-page No
Reporting of background should include		
1	Problem definition	3

2	Hypothesis statement	-
3	Description of study outcome(s)	3
4	Type of exposure or intervention used	3
5	Type of study designs used	3
6	Study population	3
Reporting of search strategy should include		
7	Qualifications of searchers (eg, librarians and investigators)	3
8	Search strategy, including time period included in the synthesis and key words	3, Add. file1
9	Effort to include all available studies, including contact with authors	3
10	Databases and registries searched	3
11	Search software used, name and version, including special features used (eg, explosion)	4
12	Use of hand searching (eg, reference lists of obtained articles)	3
13	List of citations located and those excluded, including justification	Tab. 1, Add. file 4
14	Method of addressing articles published in languages other than English	3
15	Method of handling abstracts and unpublished studies	3
16	Description of any contact with authors	3
Reporting of methods should include		
17	Description of relevance or appropriateness of studies assembled for assessing the hypothesis to be tested	4
18	Rationale for the selection and coding of data (eg, sound clinical principles or convenience)	3
19	Documentation of how data were classified and coded (eg, multiple raters, blinding and interrater reliability)	3
20	Assessment of confounding (eg, comparability of cases and controls in studies where appropriate)	4, Add. file 5
21	Assessment of study quality, including blinding of quality assessors, stratification or regression on possible predictors of study results	4, Add. file 5
22	Assessment of heterogeneity	4
23	Description of statistical methods (eg, complete description of fixed or random effects models, justification of whether the chosen models account for predictors of study results, dose-response models, or cumulative meta-analysis) in sufficient detail to be replicated	4
24	Provision of appropriate tables and graphics	Fig. 1-2, Tab. 1-2, Add. file 4-6
Reporting of results should include		
25	Graphic summarizing individual study estimates and overall estimate	Fig. 2
26	Table giving descriptive information for each study included	Tab. 1

27	Results of sensitivity testing (eg, subgroup analysis)	Tab. 2
28	Indication of statistical uncertainty of findings	6-7, Add. file 6

Item No	Recommendation	Reported on Manuscript-page No
Reporting of discussion should include		
29	Quantitative assessment of bias (eg, publication bias)	8
30	Justification for exclusion (eg, exclusion of non-English language citations)	8
31	Assessment of quality of included studies	7-8, Add. file 5
Reporting of conclusions should include		
32	Consideration of alternative explanations for observed results	7-8
33	Generalization of the conclusions (ie, appropriate for the data presented and within the domain of the literature review)	8
34	Guidelines for future research	8-9
35	Disclosure of funding source	9

From: Stroup DF, Berlin JA, Morton SC, et al, for the Meta-analysis Of Observational Studies in Epidemiology (MOOSE) Group. Meta-analysis of Observational Studies in Epidemiology. A Proposal for Reporting. *JAMA*. 2000;283(15):2008-2012. doi: 10.1001/jama.283.15.2008.

2.2 Supplementary Table 2: Reasons for exclusion

# study	Citation	Reason for exclusion
1	Andersson SI. Appraisal, coping, motivational factors and gender in vocational rehabilitation. Scand J Soc Med 1996;24(3):161–8. https://doi.org/10.1177/140349489602400307.	exposure not like defined
2	Barbek R, Makowski AC, Knesebeck O von dem. Social inequalities in health anxiety: A systematic review and meta-analysis. Journal of psychosomatic research 2021:110706. https://doi.org/10.1016/j.jpsychores.2021.110706.	publication/study type not like defined
3	Blazer, D. G., 2nd, Houpt JL. Perception of poor health in the healthy older adult. J Am Geriatr Soc 1979;27(7):330–4. https://doi.org/10.1111/j.1532-5415.1979.tb06051.x.	association measured not like defined
4	Boscarino JA. Diseases among men 20 years after exposure to severe stress: implications for clinical research and medical care. Psychosomatic Medicine 1997;59(6):605–14. https://doi.org/10.1097/00006842-199711000-00008.	association measured not like defined
5	Bowman KF, Rose JH, Deimling GT, Kypriotakis G, O'Toole EE. Primary care physicians' involvement in the cancer care of older long-term survivors. J Aging Health 2010;22(5):673–86. https://doi.org/10.1177/0898264310373501.	association measured not like defined
6	Bravo IM, Roca CS. Assessing somatoform disorders with the Hispanic client 2013:293–307. https://doi.org/10.1007/978-1-4614-4412-1_19.	publication/study type not like defined
7	Brink TL, Capri D, Neeve V de, Janakes C, Oliveira C. Senile confusion: limitations of assessment by the face-hand test, mental status questionnaire, and staff ratings. Journal of the American	outcome not like defined

	Geriatrics Society 1978;26(8):380–2. https://doi.org/10.1111/j.1532-5415.1978.tb03690.x.	
8	Brink TL, Janakes C, Martinez N. Geriatric hypochondriasis: situational factors. Journal of the American Geriatrics Society 1981;29(1):37–9. https://doi.org/10.1111/j.1532-5415.1981.tb02392.x.	outcome not like defined
9	Chang K-AJ, Kim K, Fava M, Mischoulon D, Hong JP, Kim DJH et al. Cross-national differences in hypochondriasis symptoms between Korean and American outpatients with major depressive disorder. Psychiatry Research 2016;245:127–32. https://doi.org/10.1016/j.psychres.2016.08.019.	association measured not like defined
10	Coie JD, Costanzo PR, Cox GB. Behavioral determinants of mental illness concerns: a comparison of community subcultures. Am J Community Psychol 1980;8(5):537–55. https://doi.org/10.1007/BF00912591.	outcome not like defined
11	Collings JA. International differences in psychosocial well-being: a comparative study of adults with epilepsy in three countries. Seizure 1994;3(3):183–90. https://doi.org/10.1016/s1059-1311(05)80187-6.	association measured not like defined
12	Creed F, Barsky A. A systematic review of the epidemiology of somatisation disorder and hypochondriasis. Journal of psychosomatic research 2004;56(4):391–408. https://doi.org/10.1016/S0022-3999(03)00622-6.	publication/study type not like defined
13	Deimling GT, Schaefer ML, Kahana B, Bowman KF, Reardon J. Racial Differences in the Health of Older-Adult Long-Term Cancer Survivors. J Psychosoc Oncol 2002;20(4):71–94. https://doi.org/10.1300/J077v20n04_05.	outcome not like defined
14	Demmer C. Quality of life and risk perception among predominantly heterosexual, minority individuals with HIV/AIDS. AIDS PATIENT CARE AND STDS 2001;15(9):481–9. https://doi.org/10.1089/108729101753145475.	association measured not like defined
15	D'Orazio LM, Meyerowitz BE, Korst LM, Romero R, Goodwin TM. Evidence against a link between hyperemesis gravidarum and personality characteristics from an ethnically diverse sample of pregnant women: a pilot study. J Womens Health (Larchmt) 2011;20(1):137–44. https://doi.org/10.1089/jwh.2009.1851.	association measured not like defined
16	Escobar JI, Canino G. Unexplained physical complaints. Psychopathology and epidemiological correlates. Br J Psychiatry Suppl 1989(4):24–7.	outcome not like defined
17	Fergus TA, Kelley LP, Griggs JO. The Whiteley Index-6: An Examination of Measurement Invariance Among Self-Identifying Black, Latino, and White Respondents in Primary Care. Assessment 2018;25(2):247–58. https://doi.org/10.1177/1073191116645908.	publication/study type not like defined
18	Friedman ES, Wisniewski SR, Gilmer W, Nierenberg AA, Rush AJ, Fava M et al. Sociodemographic, clinical, and treatment characteristics associated with worsened depression during treatment with citalopram: results of the NIMH STAR(*)D trial. Depress Anxiety 2009;26(7):612–21. https://doi.org/10.1002/da.20568.	association measured not like defined
19	Garza MC. A culturally sensitive psychological treatment plan for immigrant Mexican-American women diagnosed with somatic symptom disorder with predominant pain. Dissertation Abstracts International: Section B: The Sciences and Engineering 2017:No Pagination Secified.	publication/study type not like defined

20	Gogol' KN, Gotsiridze EG, Guruli ZV, Kintraia NP, Tsaava FD. The expectancy-stress factor in pregnant refugee women. Georgian Med News 2006(138):13–6.	language not like defined
21	Goodwin R, Sun S. Public perceptions and reactions to H7N9 in Mainland China. J Infect 2013;67(5):458–62. https://doi.org/10.1016/j.jinf.2013.06.014.	exposure not like defined
23	Hes JP. Hypochondriacal complaints in Jewish psychiatric patients. Isr Ann Psychiatr Relat Discip 1968;6(2):134–42.	outcome not like defined
22	Hes JP. HYPOCHONDRIASIS IN ORIENTAL JEWISH IMMIGRANTS. Int J Soc Psychiatry 1958;4(1):18–23. https://doi.org/10.1177/002076405800400103.	association measured not like defined
24	Hogg JLC. Impact of personality on communication: An MMPI-2 study of African American college students and their choice in the digital communications age. Dissertation Abstracts International: Section B: The Sciences and Engineering 2010:2092.	publication/study type not like defined
25	Hunter LR, Schmidt NB. Anxiety psychopathology in African American adults: literature review and development of an empirically informed sociocultural model. Psychological Bulletin 2010;136(2):211–35. https://doi.org/10.1037/a0018133.	outcome not like defined
26	Jenkins CD. Psychologic and social precursors of coronary disease (first of two parts). N Engl J Med 1971;284(5):244–55. https://doi.org/10.1056/NEJM197102042840506.	exposure not like defined
27	Landau SF, Beit-Hallahmi B, Levy S. Personal and the political: Israelis' perception of well-being in times of war and peace. SOCIAL INDICATORS RESEARCH 1998;44(3):329–65. https://doi.org/10.1023/A:1006885502825.	outcome not like defined
28	Larbig W, Xenakis C, Onishi MS. Psychosomatic symptoms and functional disorders in migrant workers--Japanese and Greeks in Germany and Germans abroad. Z Psychosom Med Psychoanal 1979;25(1):49–63.	outcome not like defined
29	Liddell BJ, O'Donnell M, Bryant RA, Murphy S, Byrow Y, Mau V et al. The association between COVID-19 related stressors and mental health in refugees living in Australia. Eur J Psychotraumatol 2021;12(1):1947564. https://doi.org/10.1080/20008198.2021.1947564.	association measured not like defined
30	Lim MC, Shiba DR, Clark IJ, Kim DY, Styles DE, Brandt JD et al. Personality type of the glaucoma patient. J Glaucoma 2007;16(8):649–54. https://doi.org/10.1097/IJG.0b013e31806ab2eb.	association measured not like defined
31	Maharaj R, Alexander C, Bridglal CH, Edwards A, Mohammed H, Rampaul T et al. Somatoform disorders among patients attending walk-in clinics in Trinidad: prevalence and association with depression and anxiety. Ment Health Fam Med 2013;10(2):81–8.	outcome not like defined
32	Mechanic D. Social psychologic factors affecting the presentation of bodily complaints. N Engl J Med 1972;286(21):1132–9. https://doi.org/10.1056/NEJM197205252862105.	outcome not like defined
33	Minc, S. OF NEW AUSTRALIAN PATIENTS, THEIR MEDICAL LORE AND MAJOR ANXIETIES. undefined 1963.	publication/study type not like defined
34	Mumford DB, Bavington JT, Bhatnagar KS, Hussain Y, Mirza S, Naraghi MM. The Bradford Somatic Inventory. A multi-ethnic inventory of somatic symptoms reported by anxious and depressed patients in Britain and the Indo-Pakistan subcontinent. Br J Psychiatry 1991;158:379–86. https://doi.org/10.1192/bjp.158.3.379.	outcome not like defined
35	Novaković M, Ile T, Marić-Tiosavljević D, Munzić I. Suicidal and parasuicidal behaviour. Med Arh 2006;60(1):44–8.	language not like defined

36	Novakovic M, Licanin I, Musić E, Novaković R, Jovanović D, Frasto E. Somatisation disorder in nephrologic patients. Med Arh 2008;62(1):25–9.	association measured not like defined
37	O'Connor EA, Grunert BK, Matloub HS, Eldridge MP. Factitious hand disorders: review of 29 years of multidisciplinary care. J Hand Surg Am 2013;38(8):1590–8. https://doi.org/10.1016/j.jhsa.2013.04.047.	association measured not like defined
38	Ottenhoff JSE, Kortlever JTP, Boersma EZ, Laverty DC, Ring D, Driscoll MD. Adverse Childhood Experiences Are Not Associated With Patient-reported Outcome Measures in Patients With Musculoskeletal Illness. Clinical Orthopaedics and Related Research 2019;477(1):219–28. https://doi.org/10.1097/CORR.0000000000000519.	association measured not like defined
39	Park CL, Cho D, Blank TO, Wortmann JH. Cognitive and emotional aspects of fear of recurrence: predictors and relations with adjustment in young to middle-aged cancer survivors. Psycho-oncology 2013;22(7):1630–8. https://doi.org/10.1002/pon.3195.	outcome not like defined
40	Pascual-Vera B, Akin B, Belloch A, Bottesi G, Clark DA, Doron G et al. The cross-cultural and transdiagnostic nature of unwanted mental intrusions. Int J Clin Health Psychol 2019;19(2):85–96. https://doi.org/10.1016/j.ijchp.2019.02.005.	association measured not like defined
41	Pilowsky I. Dimensions of abnormal illness behaviour. Aust N Z J Psychiatry 1975;9(3):141–7. https://doi.org/10.3109/00048677509159839.	association measured not like defined
42	Poeck K. Hypochondriac depressions due to uprooting in Italian workers in Germany. Dtsch Med Wochenschr 1962;87:1419–24. https://doi.org/10.1055/s-0028-1112083.	association measured not like defined
43	Portelli AJ, Jones I. "Mediterranean guts ache". Med J Aust 1969;2(14):717–20. https://doi.org/10.5694/j.1326-5377.1969.tb107354.x.	publication/study type not like defined
44	Saint EG. The medical problems of migrants. Med J Aust 1963;50(1):335–8. https://doi.org/10.5694/j.1326-5377.1963.tb23062.x.	outcome not like defined
45	Schatz D, Harder D, Schatz M, Harden K, Chilingar L, Fox D et al. The relationship of maternal personality characteristics to birth outcomes and infant development. Birth 2000;27(1):25–32. https://doi.org/10.1046/j.1523-536x.2000.00025.x.	association measured not like defined
46	Simning A, Seplaki CL, Conwell Y. The association of an inability to form and maintain close relationships due to a medical condition with anxiety and depressive disorders. Journal of affective disorders 2016;193:130–6. https://doi.org/10.1016/j.jad.2015.12.079.	outcome not like defined
47	Slesinger D, Archer RP, Duane W. MMPI-2 characteristics in a chronic pain population. Assessment 2002;9(4):406–14. https://doi.org/10.1177/1073191102238153.	association measured not like defined
48	Stone AJ, Siegel JM. Correlates of accurate knowledge of cancer. Health Educ Q 1986;13(1):39–50. https://doi.org/10.1177/109019818601300105.	outcome not like defined
49	Suvinen TI, Reade PC, Sunden B, Gerschman JA, Koukounas E. Temporomandibular disorders: Part II. A comparison of psychologic profiles in Australian and Finnish patients. J Orofac Pain 1997;11(2):147–57.	association measured not like defined
50	Verschuur MJ, Maric M, Spinhoven P. Differences in changes in health-related anxiety between Western and non-Western participants in a trauma-focused study. Journal of traumatic stress 2010:300–3.	outcome not like defined

51	Wenzel LB, Anderson R, Tucker DC, Palla S, Thomson E, Speechley M et al. Health-related quality of life in a racially diverse population screened for hemochromatosis: results from the Hemochromatosis and Iron Overload Screening (HEIRS) study. Genet Med 2007;9(10):705–12. https://doi.org/10.1097/gim.0b013e3181571f31.	outcome not like defined
52	Woo M, Oei TPS. MMPI-2 profiles of Australian and Singaporean psychiatric patients. Psychiatry Research 2007;150(2):153–61. https://doi.org/10.1016/j.psychres.2006.04.007.	association measured not like defined

2.3 Supplementary Table 3: Quality assessment assessed with the Effective Public Health Practice Project Quality Assessment Tool (EPHPP)

	Selection bias	Study design	Confounders	Data collection	Withdrawls/ dropouts	Statistical analysis	Total score
Akariya et al. (2021)	-	+	++	++	x	++	moderate
Barsky (1998)	+	++	-	++	+	-	weak
Barsky et al. (1990)	+	+	-	++	x	-	weak
Bhatt et al. (1989)	+	+	-	++	x	-	weak
Bravo&Arrufat (2005)	-	++	-	++	x	+	weak
Escobar (1998)	-	+	-	++	x	-	weak
Fergus et al. (2017)	+	+	+	++	x	+	moderate
Gerdes&Noyes (1996)	+	+	-	++	x	-	weak
Goel et al. (2002)	-	+	+	++	x	+	moderate
Hollifield (1999)	+	+	-	++	x	-	weak
Kibbey et al. (2021)	-	+	+	++	x	+	moderate
Looper et al. (2001)	+	+	-	++	x	-	weak
Mölsä et al. (2014)	++	++	-	++	x	-	weak
Noyes (2005)	-	+	-	++	x	-	weak
Noyes et al. (2004)	-	++	+	++	-	++	weak
Noyes (1999)	+	+	-	++	x	-	weak
Pilowsky&Spence (1977)	+	+	-	++	x	+	moderate
Pine (1983)	-	++	-	++	x	+	weak

Strength of evidence	++ strong	+ moderate	- weak	x not applicable

2.4 Supplementary Figure 1: Funnel plot (trim-and-fill method)

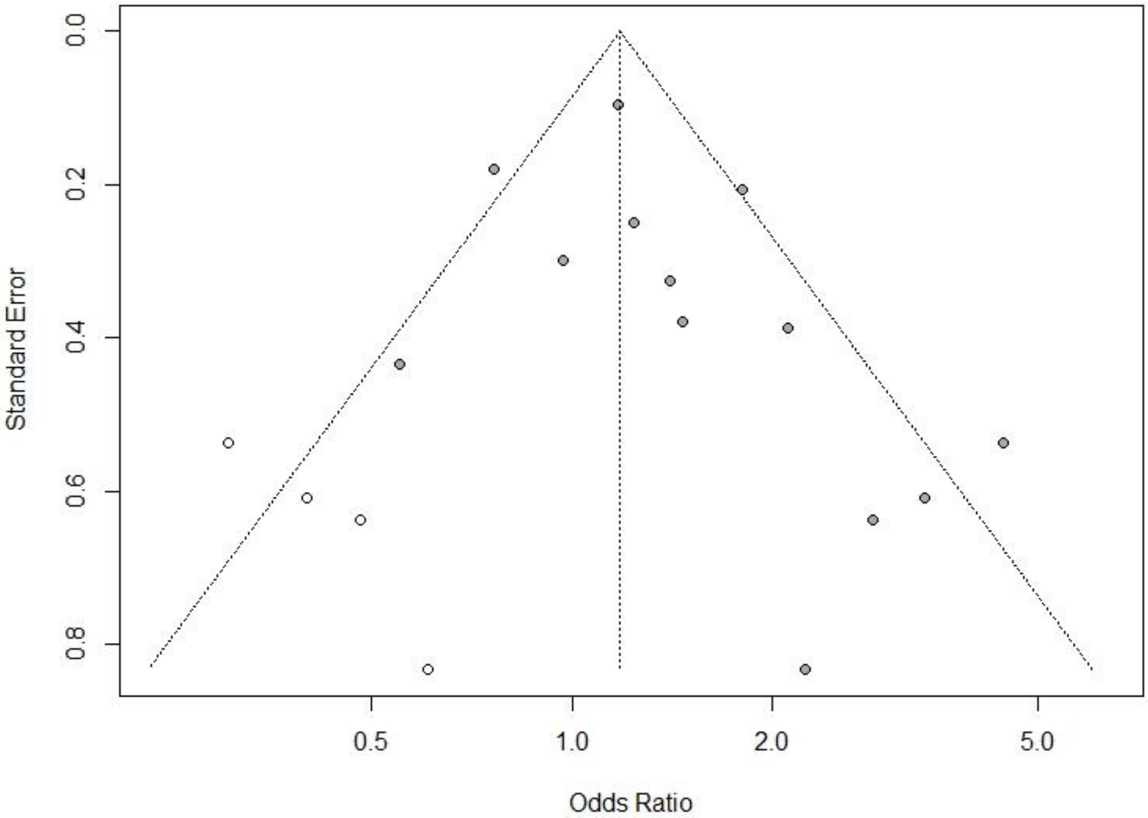

Supplement: Supplementary file 1 [file Data_Sheet_1.pdf]
